# Identification of Prostate-Specific G-Protein Coupled Receptor as a Tumor Antigen Recognized by CD8^+^ T Cells for Cancer Immunotherapy

**DOI:** 10.1371/journal.pone.0045756

**Published:** 2012-09-20

**Authors:** Satoko Matsueda, Mingjun Wang, Jinsheng Weng, Ying Li, Bingnan Yin, Jia Zou, Qingtian Li, Wei Zhao, Weiyi Peng, Xavier Legras, Christopher Loo, Rong-Fu Wang, Helen Y. Wang

**Affiliations:** 1 Center for Cell and Gene Therapy, Baylor College of Medicine, Houston, Texas, United States of America; 2 Center for Inflammation and Epigenetics, The Methodist Hospital Research Institute, Houston, Texas, United States of America; 3 Department of Pathology and Immunology, Baylor College of Medicine, Houston, TX, United States of America; Johns Hopkins University, United States of America

## Abstract

**Background:**

Prostate cancer is the most common cancer among elderly men in the US, and immunotherapy has been shown to be a promising strategy to treat patients with metastatic castration-resistant prostate cancer. Efforts to identify novel prostate specific tumor antigens will facilitate the development of effective cancer vaccines against prostate cancer. Prostate-specific G-protein coupled receptor (PSGR) is a novel antigen that has been shown to be specifically over-expressed in human prostate cancer tissues. In this study, we describe the identification of PSGR-derived peptide epitopes recognized by CD8^+^ T cells in an HLA-A2 dependent manner.

**Methodology/Principal Findings:**

Twenty-one PSGR-derived peptides were predicted by an immuno-informatics approach based on the HLA-A2 binding motif. These peptides were examined for their ability to induce peptide-specific T cell responses in peripheral blood mononuclear cells (PBMCs) obtained from either HLA-A2^+^ healthy donors or HLA-A2^+^ prostate cancer patients. The recognition of HLA-A2 positive and PSGR expressing LNCaP cells was also tested. Among the 21 PSGR-derived peptides, three peptides, PSGR3, PSGR4 and PSGR14 frequently induced peptide-specific T cell responses in PBMCs from both healthy donors and prostate cancer patients. Importantly, these peptide-specific T cells recognized and killed LNCaP prostate cancer cells in an HLA class I-restricted manner.

**Conclusions/Significance:**

We have identified three novel HLA-A2-restricted PSGR-derived peptides recognized by CD8^+^ T cells, which, in turn, recognize HLA-A2^+^ and PSGR^+^ tumor cells. The PSGR-derived peptides identified may be used as diagnostic markers as well as immune targets for development of anticancer vaccines.

## Introduction

Prostate cancer has become the most common cancer among men in the US and is the second leading cause of death from cancer in American men [1]. The standard of care for most patients with prostate cancer is surgery and/or radiation therapy. However, disease recurrence after surgery or radiation still takes place in up to 30% of patients. Although androgen-deprivation therapy is an effective treatment against recurrent disease, most of these patients eventually develop androgen-refractory prostate cancer, which is insensitive to traditional treatment. Therefore, more effective and less toxic therapies are urgently needed. Immunotherapy has been shown to be a promising approach to the treatment of prostate cancer, especially for patients with metastatic castration-resistant prostate cancer [2]–[4]. Harnessing the immune system to eradicate malignant cells is a promising approach for cancer therapy, but until recently it has been met with only sporadic clinical success [4]–[6]. Recent Food and Drug Administration (FDA) approvals of the immunotherapy-based vaccine/drug sipuleucel-T *(*Provenge) and ipilimumab (Yervoy) represent milestones in the field of cancer immunotherapy [7], [8]. Furthermore, a phase III clinical trial of the gp100 peptide for melanoma also produced highly encouraging clinical results [9]. However, the clinical benefits reported for these agents have fallen far short of complete responses and permanent cures. In the case of sipuleucel-T, the survival benefit for patients was only 4.1 months, without objective tumor regression or substantial changes in prostate specific antigen (PSA) levels. A recent study using animal models further reveals the importance of tumor-specific antigens in eliciting immune responses against a developing tumor [10], spurring more efforts to identify such antigens for cancer immunotherapy. Furthermore, since some major rejection antigens may be lost or altered due to T cell selection and killing [11], the best strategy is to target multiple tumor antigens that are present on individual tumors for immunotherapy.

To date, a number of prostate specific tumor antigens have been well-defined, including PSA [12], [13], prostein [14], [15], prostate stem cell antigen (PSCA) [16], prostate-specific membrane antigen (PSMA) [17]–[19], prostatic acid phosphatase (PAP) [20] and transient receptor potential p8 (trp-p8) [21]. Furthermore, HLA-class I-restricted epitopes derived from these tumor antigens have been described [22]. One drawback of single tumor antigen-based immunotherapy is that immune escape may occur. Hence, identification of additional prostate cancer-specific antigens for development of more effective and antigen-specific vaccines for patients with metastatic prostate cancer is needed. Prostate-specific G-protein coupled receptor (PSGR) is a member of the G-protein coupled odorant receptor family, and is highly expressed in prostate cancer cells compared with normal prostate cells [23]–[25], suggesting that PSGR may be targeted for the development of novel immunotherapeutic strategies against prostate cancer.

In an attempt to determine whether PSGR can be recognized by T cells, we describe the identification of PSGR-derived T cell epitopes for T cell recognition by an immuno-bioinformatics approach. Twenty-one peptides that were predicted to bind to the HLA-A2 molecule were selected and synthesized. All these peptides were evaluated *in vitro* for their ability to stimulate T cells in PBMCs from both healthy subjects and prostate patients based on interferon-γ (IFN-γ) release measured by ELISA or ELISPOT assays. Three peptides were found to induce IFN-γ release in peripheral T cells from both healthy subjects and prostate cancer patients. Importantly, these peptide-specific T cells could recognize HLA-A2^+^, PSGR-expressing LNCaP cells in an HLA-class I-dependent manner.

## Materials and Methods

### Healthy Donors and Prostate Cancer Patients

Ten HLA-A2^+^ prostate cancer patients and ten HLA-A2^+^ healthy subjects were enrolled in this study after written informed consent was obtained. All protocols were approved by the Institutional Review Board (IRB) of Baylor College of Medicine before commencing studies. 20 mL of peripheral blood was obtained from each person, and peripheral blood mononuclear cells (PBMCs) were isolated by density gradient centrifugation using Lymphoprep (Nycomed Pharma AS; Oslo, Norway). The freshly isolated PBMCs were cryopreserved for later use in 1 mL freezing medium containing 90% FCS and 10% dimethyl sulfoxide (DMSO) at −140°C. The expression of HLA-A2 molecules on PBMCs obtained from cancer patients and healthy subjects was verified by flow cytometry with FITC-labeled HLA-A2 mAb BB7.2 (BD Pharmingen, San Diego, CA, USA).

### Cell Lines

T2 cells (an HLA-A2^+^ TAP-deficient cell line), PC3 cells (an HLA-A2-negative prostate cancer cell line), and LNCaP cells (an HLA-A2 positive prostate carcinoma cell line) were all purchased from American Type Culture Collection (ATCC; Manassas, VA, USA). All cell lines were maintained in RPMI-1640 medium (Mediatech; Manassas, VA, USA), supplemented with 10% FBS, 1% L-glutamine, and 1% penicillin and streptomycin.

### Peptides

Twenty-one PSGR-derived peptides (Table 1) were predicted using BIMAS (http://www-bimas.cit.nih.gov/molbio/hla_bind/), SYFPEITHI (http://www.syfpeithi.de/), and Rankpep (http://bio.dfci.harvard.edu/Tools/rankpep.html) based on the HLA-A2 binding motif. Only epitopes that were predicted by at least two of these algorithms were selected for further testing. The peptides were synthesized by a solid-phase method using a peptide synthesizer (AApptec, Inc.; Louisville, KY, USA), purified by reverse-phase high-performance liquid chromatography and validated by mass spectrometry. The synthesized peptides were dissolved in DMSO at a concentration of 10 mg/mL and stored at −80°C until further use.

**Table 1:** The predicted HLA-A2 binding peptides derived from the prostate-specific G-protein coupled receptor (PSGR).

Peptide #	HLA restriction	Position	Sequence
PSGR1	HLA-A2	287–295	VLNPIVYGV
PSGR2	HLA-A2	188–196	KLACDDIRV
PSGR3	HLA-A2	276–284	ILANIYLLV
PSGR4	HLA-A2	28–36	WLAFPLCSL
PSGR5	HLA-A2	220–228	YLLILKTVL
PSGR7	HLA-A2	181–189	CLHQDVMKL
PSGR10	HLA-A2	213–222	SLLISFSYLL
PSGR11	HLA-A2	245–254	HVCAVFIFYV
PSGR12	HLA-A2	21–30	GLEEAQFWLA
PSGR13	HLA-A2	156–165	ALMAPLPVFI
PSGR14	HLA-A2	275–284	VILANIYLLV
PSGR15	HLA-A2	221–230	LLILKTVLGL
PSGR16	HLA-A2	37–46	YLIAVLGNLT
PSGR17	HLA-A2	66–75	CMLSGIDILI
PSGR18	HLA-A2	100–109	LLQMFAIHSL
PSGR19	HLA-A2	56–65	SLHEPMYIFL
PSGR20	HLA-A2	117–126	LLAMAFDRYV
PSGR21	HLA-A2	41–50	VLGNLTIIYI
PSGR22	HLA-A2	250–259	FIFYVPFIGL
PSGR23	HLA-A2	139–148	TLPRVTKIGV
PSGR24	HLA-A2	253–262	YVPFIGLSMV

### *In vitro* Stimulation of Peptide-specific T cells in PBMCs

PBMCs (1×10^5^ cells/well) from either healthy subjects or prostate cancer patients were incubated with standard peptide concentrations of 20 µg/mL per peptide [26]–[28] in 96-well U-bottom microplates (BD, Franklin Lakes, NJ, USA) in 200 µL of T-cell medium (TCM), consisting of RPMI 1640 (Mediatech, Manassas, VA, USA), 10% human AB serum (Valley Biomedical, Winchester, USA), 50 µM of 2-mercaptoethanol, 100 U/mL of interleukin-2 (IL-2), and 0.1 mM MEM nonessential amino acid solution (Invitrogen, Grand Island, NY, USA). Half of the TCM was removed and replaced with fresh TCM containing peptides (20 µg/mL) every 5 days. After 14 days of culture, the cells were harvested and tested for their ability to produce IFN-γ in response to T2 cells (1×10^4^ cells/well), which were pre-loaded with either PSGR peptide (5 µg/mL) or a control peptide (an irrelevant HLA-A2 binding peptide: NLLTHVESL ) as a negative control. After 18 hours of incubation, supernatants were collected, and IFN-γ release was determined by ELISA assay.

### Rapid Expansion Protocol (REP) for PSGR Peptide-specific T-cells

PSGR peptide specific T cells were expanded by a rapid expansion protocol (REP) as previously described [29] with a slight modification. Briefly, on day 0, 0.1−0.5×10^6^ PSGR peptide specific T cells were cultured in a T25 flask with 20 mL RPMI-1640 supplemented with 10% human AB serum, 50 µM of 2-mercaptoethanol, and 30 ng/mL OKT3 antibody (Ortho Biotech, Bridgewater, NJ), together with 20×10^6^ irradiated allogeneic PBMCs and 5×10^6^ irradiated Epstein Barr Virus (EBV) transformed B-cells as feeder cells. Flasks were incubated upright at 37°C in 5% CO_2_. IL-2 (300 IU/mL) was added on day 1, and on day 5, half of the cell culture supernatant was removed and replenished with fresh medium containing 300 IU/mL IL-2. 14 days after initiation of the REP, cells were harvested and cryopreserved for future experiments.

### ELISA Assay

Cytokine release was measured by coating 96-well ELISA plates (Thermo Fisher Scientific; Rochester, NY, USA) with 1 µg/mL anti-human IFN-γ (Pierce Biotechnology; Rockford, IL, USA) overnight at 4°C. The plate was washed six times with PBS containing 0.05% Tween-20 (wash solution) to remove unbound coating antibody, and blocked with 1% BSA/PBS at room temperature for 2 hrs. Afterwards, 50 µL supernatant was added to each well and incubated at room temperature for 1 hr, then 50 µL of 0.5 µg/mL biotinylated anti-human IFN-γ (Pierce Biotechnology; Rockford, IL, USA) was added and plates were incubated for an additional 1 hr at room temperature. After incubation, plates were washed and incubated for 30 min with Poly-HRP-Streptavidin (Thermo Fisher Scientific; Rochester, NY, USA) diluted 1∶5000 in PBS/1% BSA. Plates were washed and 100 µL of TMB substrate solution (Sigma-Aldrich Co.; St. Louis, MO, USA) was added per well. The colorimetric reaction was stopped using 2N H_2_SO4 and plates were read at 450 nm using an ELISA plate reader.

### IFN-γ ELISPOT Assay

The IFN-γ ELISPOT assay was performed as previously described [27] to quantify peptide-specific cytotoxic T-lymphocytes (CTLs) after *in vitro* expansion. Briefly, 96-well ELISPOT plates (Millipore; Bedford, MA, USA) were coated overnight at 4°C with 7.5 µg/mL anti-human IFN-γ (Pierce Biotechnology; Rockford, IL, USA). Plates were washed six times with sterile PBS to remove unbound coating antibody. T cells were seeded at 1×10^5^ cells per well and incubated with T2 cells alone, T2 cells pulsed with a PSGR peptide (5 µg/mL) or an irrelevant peptide as a negative control. Cells stimulated with 5 µg/mL OKT3 antibody (Ortho Biotech; Bridgewater, NJ, USA) were used as positive control. After incubating samples for 18 - 20 hrs at 37°C and 5% CO_2_, plates were washed with wash solution. 0.75 µg/mL Biotinylated anti-human IFN-γ (Pierce Biotechnology; Rockford, IL, USA) was added, and plates were incubated for 2 hrs at room temperature. After incubation, plates were washed with wash solution and incubated further with Poly-HRP-Streptavidin (Thermo Fisher Scientific; Rochester, NY, USA) diluted 1∶1000 in PBS/1% BSA for 1 hr. Plates were washed and 200 µL of 4-chloro-1-naphthol substrate (Sigma-Aldrich Co.; St. Louis, MO, USA) was added to each well. Finally, plates were washed under running tap water and dried at room temperature. IFN-γ spot-forming cells (SFC) were enumerated using an ELISPOT reader (C.T.L. Technologies, Minneapolis, MN, USA).

### RNA Extraction and RT-PCR

RNA extraction and RT-PCR was carried out as reported previously [30]. In brief, total RNA was extracted from prostate cancer cells with 1 mL Trizol reagent (Invitrogen; Carlsbad, CA, USA). Three micrograms of RNA was reverse-transcribed to cDNA in 30 µl volume and 1 µl of each cDNA was used in subsequent PCR reaction with a pair of PSGR specific primers: Primer 1: 5′-GAAGATCTATGAGTTCCTGCAACTTC-3′, primer 2: 5′-CCCAAGCTT TCACTTGCCTCCCACAG-3′. β-actin was used as loading control: primer 1: 5′-CATGATGGAGTTGAAGGTAGTTTCG-3′; Primer 2: 5′-CAGACTATGCTGTCCCTGTACGC-3′. The PCR reaction was carried out under the following conditions: 94°C for 2 min, 94°C for 30 s, 56°C for 30 s, 72°C for 1 min 20 s, total 35 cycles, 72°C for 10 min, and β-actin was run for 25 cycles. Equal amounts of PCR products were then loaded and detected by gel electrophoresis.

### Cytotoxicity Assay

PSGR derived peptide-specific T cells were tested for cytotoxicity against both PC3 and LNCaP by a lactate dehydrogenase (LDH) assay (Promega; Madison, WI, USA). The assay was performed in accordance with the manufacturer’s instructions. LDH release was calculated based on the following formula:

Cytotoxicity (%)  =  (Experimental – Effector Spontaneous – Target Spontaneous LDH release)/(Target Maximum – Target Spontaneous LDH release) × 100.

Spontaneous release was determined by using the supernatant of the target cells alone or effector cells alone, and the maximum release was determined by using the supernatant of target cells incubated with a lysis solution included in LDH kit. To determine if T cell recognition is HLA-I restricted, anti-HLA-I, anti-HLA-II, or anti-CD19 mAb (all from ATCC, Manassas, VA, USA) were added into wells at the initiation of the culture.

### Intracellular IFN-γ Cytokine Staining

PSGR-derived peptide specific T cells (0.5−1×10^6^ ) were cultured with 0.5×10^6^ T2 cells pulsed with or without peptide (5 µg/mL) in the presence of GolgiStop (BD Pharmingen, San Diego, CA, USA) in a 48-well plate for 4 hrs at 37°C. Cells were stained with anti-CD8 and anti-IFN-γ and analyzed using a FACScalibur machine.

### Statistics

Student’s t-test was used to analyze quantitative differences between the experimental wells and controls in ELISA and ELISPOT assays. P<0.05 was considered significant.

## Results

### Induction of PSGR Derived Peptide-specific CTLs in Healthy Donors

To determine whether PSGR-reactive T cell precursors are present in healthy subjects, we obtained PBMCs from 10 HLA-A2^+^ healthy donors and stimulated them *in vitro* with each of the 21 PSGR-derived peptides containing HLA-A2-binding motif (Table 1). After 2 weeks of peptide stimulation, supernatants from peptide-stimulated T cells were analyzed by ELISA assay to detect IFN-γ release in response to T2 cells pulsed with or without corresponding peptides. As shown in Table 2, 13 PSGR-derived peptides were capable of inducing peptide-specific T-cell responses in at least one of 10 healthy subjects. Importantly, PSGR3, PSGR4 and PSGR14 could induce T cell responses in 7 out of 10 healthy subjects, indicating that these 3 peptides are immunogenic and potentially capable of expanding antigen-specific T cells in healthy subjects.

**Table 2:** Induction of peptide-specific T cells from the PBMCs of ten HLA-A2^+^ healthy subjects.

	#1	#2	#3	#4	#5	#6	#7	#8	#9	#10
PSGR1	195	0	0	0	0	281	–	–	–	–
PSGR2	0	0	455	0	0	0	–	–	–	–
**PSGR3**	**127**	**398**	**393**	**768**	**227**	**183**	**645**	**0**	**394**	**0**
**PSGR4**	**302**	**152**	**457**	**0**	**407**	**226**	**847**	**0**	**0**	**459**
PSGR5	0	0	0	0	0	0	–	–	–	–
PSGR7	0	0	0	0	168	0	–	–	–	–
PSGR10	917	0	0	0	0	0	–	–	–	–
PSGR11	0	0	0	0	0	0	–	–	–	–
PSGR12	0	139	0	246	0	432	–	–	–	–
PSGR13	0	0	0	0	0	0	–	–	–	–
**PSGR14**	**471**	**0**	**115**	**0**	**124**	**296**	**843**	**0**	**346**	**854**
PSGR15	0	0	456	0	0	0	–	–	–	–
PSGR16	602	0	0	0	163	0	–	–	–	–
PSGR17	0	0	0	0	0	0	–	–	–	–
PSGR18	176	0	0	0	0	0	–	–	–	–
PSGR19	0	0	0	0	195	0	–	–	–	–
PSGR20	0	0	0	0	0	0	–	–	–	–
PSGR21	0	291	0	0	300	377	–	–	–	–
PSGR22	289	0	0	0	0	0	–	–	–	-
PSGR23	0	0	0	0	0	0	–	–	–	–
PSGR24	0	0	0	0	0	0	–	–	–	–

**Note:** Values denote concentrations of IFN-γ (pg/ml) in the supernatants; –, not done.

### Presence of PSGR-derived Peptide Specific CTLs in Prostate Cancer Patients

Since peptide-specific T cells against PSGR3, PSGR4 and PSGR14 were found in more than 70% of healthy subjects, we reasoned that CTL precursors that recognize these 3 peptides may also be high in PBMCs of prostate cancer patients. To test our hypothesis, we examined whether these three peptide candidates can induce peptide-specific CTLs from the PBMCs of HLA-A2^+^ prostate cancer patients. PBMCs from prostate cancer patients were collected and stimulated *in vitro* with PSGR3, PSGR4 or PSGR14 peptides. As shown in Table 3, PSGR3, PSGR4 and PSGR14 indeed induced peptide-specific CTLs from PBMCs of prostate cancer patients.

**Table 3:** Induction of peptide-specific T cells from the PBMCs of HLA-A2^+^ prostate cancer patients.

	#2	#3	#12	#13	#14	#15	#16	#17	#22	#25
PSGR3	525	503	326	217	0	282	35	156	103	22
PSGR4	451	351	0	301	309	70	6.7	102	56	27
PSGR14	344	116	272	23	0	24	0	100	8	11

**Note:** Values denote concentrations of IFN-γ (pg/ml) in the supernatants.

### Recognition of Prostate Cancer Cell Lines by the PSGR Derived Peptide-specific T cells

**Figure 1:**
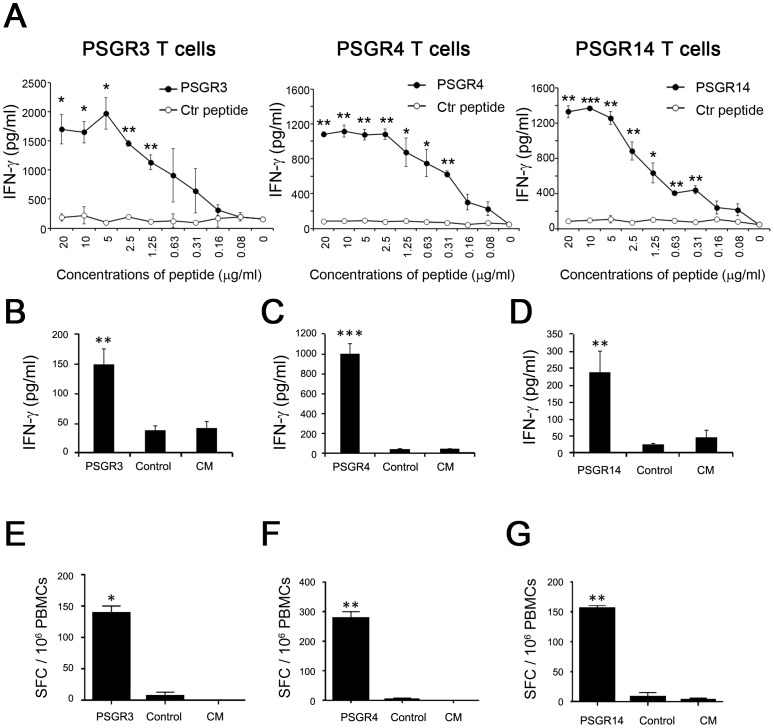
PSGR-derived peptides induced peptide-specific T cells. The recognition of T2 cells pre-loaded with titrated concentrations of peptides (0–20 µg/ml) by expanded PSGR peptide-specific T cells was tested by ELISA assay (A). The expanded PSGR3 T cells (B and E), PSGR4 T cells (C and F) and PSGR14 T cells (D and G) were respectively co-incubated with T2 cells (1×10^4^ cells/well) alone in complete medium (CM), or with T2 cells pre-loaded with either a corresponding peptide (5 µg/mL) or a control peptide as a negative control. Cells were incubated for 18 −24 hours, the IFN-γ secretion in the supernatant was determined by ELISA assay (B, C and D). IFN-γ spot-forming cells (SFC) were enumerated by ELISPOT assay (E, F and G). Data are plotted as means ± SD. Results are representative of at least three independent experiments. *P<0.05, **P<0.01, ***P<0.001 versus controls (T2 cells alone or T2 cells pulsed with a control peptide).

To obtain a large number of PSGR peptide-specific T cells for further analysis, we expanded PSGR peptide-specific T cells identified in Tables 2 and 3. To determine an effective concentration of peptide for loading T2 cells for T cell recognition, we performed peptide titration experiments. As shown in Figure 1A, for all 3 peptides a peptide concentration of 5 µg/ml was sufficient to saturate the binding sites of HLA-A2 molecules on T2 cells for T cell recognition. Therefore, we consistently used this peptide concentration for pre-loading T2 cells in ELISA and/or ELISPOT assays. The expanded T cells maintained antigen-specificity and secreted significant amounts of IFN-γ after stimulation with T2 cells pulsed with the corresponding peptides, but not with a control peptide (Figures 1A, B, C, D). ELISPOT assay further confirmed the presence of PSGR peptide-specific T cells in expanded T cells (Figures 1E, F, G)

**Figure 2:**
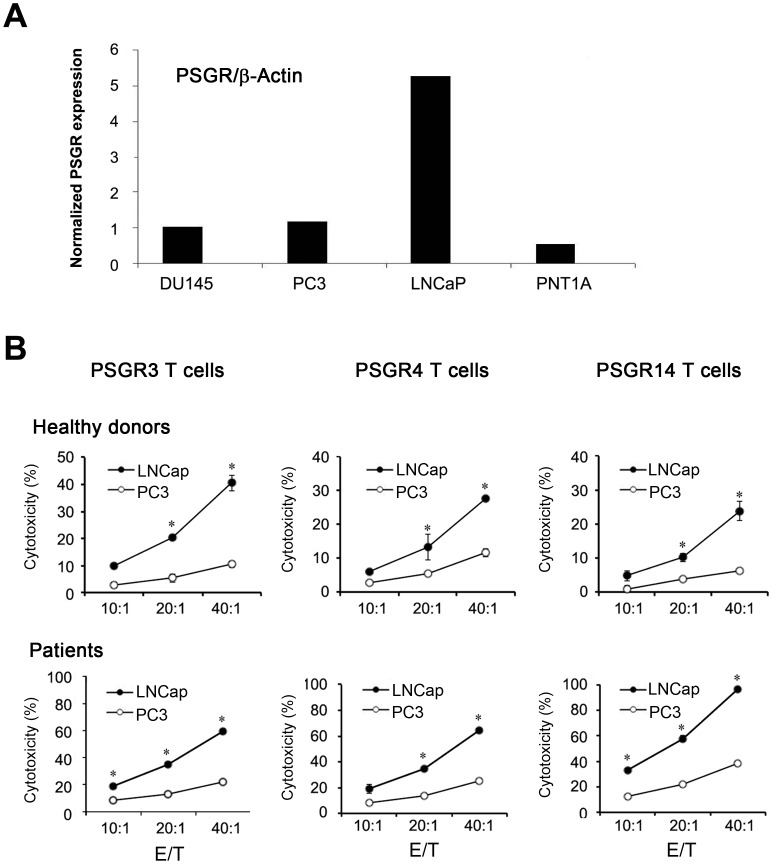
PSGR-derived peptide-specific T cells recognized HLA-A2 positive PSGR-expressing LNCaP prostate cancer cells. The expression of PSGR mRNA in different cell lines was determined by RT-PCR (A). PSGR derived peptide-specific T cells were tested for cytotoxicity against both PC3 and LNCaP by the LDH assay (B). Data from B are plotted as means ± SD. Results are representative of three independent experiments. *P<0.05, versus control.

To determine whether PSGR-derived peptide-specific T cells were able to recognize and kill HLA-A2^+^, PSGR-expressing prostate cancer cells, we used an HLA-A2 negative PC3 cell line and an HLA-A2 positive LNCaP prostate cancer cell line. The expression of PSGR in these two cell lines was examined by RT-PCR. Consistent with a previous report [31], PSGR was highly expressed in LNCaP, but not in PC3, DU145 or a normal prostate cell line PNT1A (Figure 2 A). As shown in Figure 2B, PSGR3-, PSGR4-, or PSGR14-specific T cells from both healthy donors and patients could recognize and kill HLA-A2 positive, PSGR expressing LNCaP, but not HLA-A2 negative PC3 cells. These results suggest that PSGR-specific T cells recognize T cell epitopes that are endogenously processed and presented by prostate tumor cells.

### T Cells Recognize PSGR-derived Peptides in an HLA-I Restricted Manner

**Figure 3:**
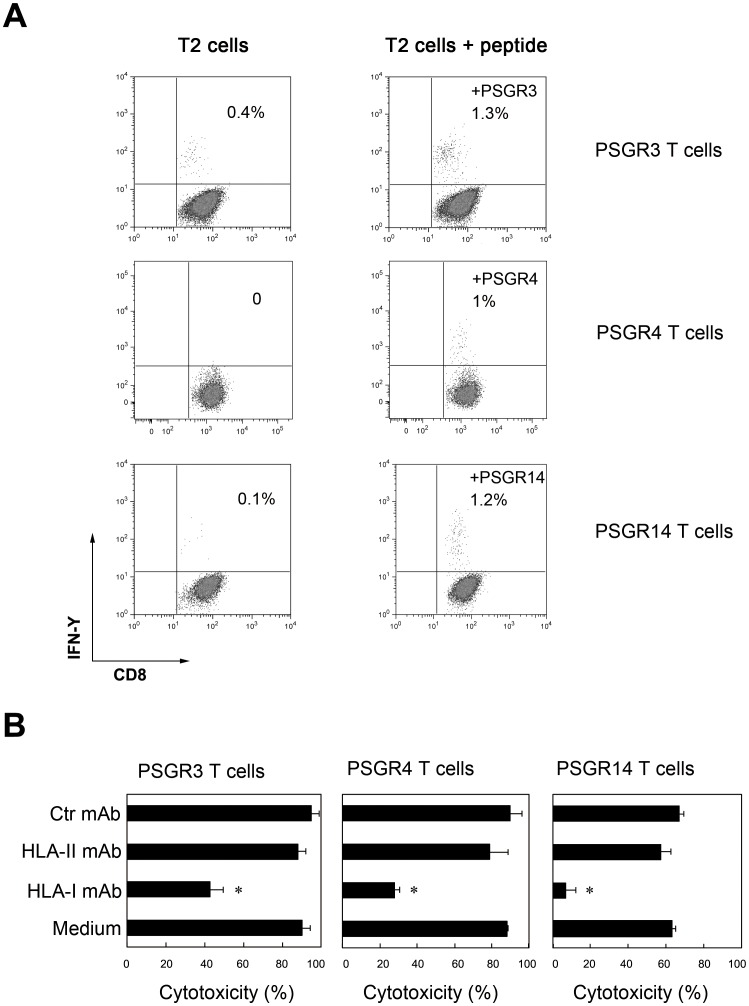
PSGR-derived peptide-induced T cell responses were CD8^+^ T cell dependent and restricted by HLA-I. PSGR-derived peptide-specific T cells were co-cultured with T2 cells pulsed with or without a given peptide in the presence of GolgiStop in a 48-well plate for 4 hrs at 37°C. Cells were stained with anti-CD8 and anti-IFN-γ, then analyzed on a FACScalibur machine (A). PSGR-derived peptide-specific T cells were co-incubated with LNCaP cells alone in medium, or with LNCaP cells in the presence of either anti-HLA-I mAb (W6/32), HLA-II mAb or a control mAb (anti-CD19 mAb). After 4 hours of incubation, the cytotoxicity against LNCaP was determined by the LDH assay (B). Data from B are plotted as means ± SD. Results are representative of three independent experiments. *P<0.05, versus controls.

To test whether the responses induced by PSGR-derived peptides are dependent on CD8^+^ T cells, we co-cultured PSGR-derived peptide-specific T cells with T2 cells pulsed with or without corresponding peptides in the presence of GolgiStop for 4 h. Staining for CD8 molecules and intracellular IFN-γ was subsequently performed. Only CD8^+^ T cells were found to produce IFN-γ in response to T2 cells pulsed with corresponding peptides (Figure 3A), while CD4^+^ T cells did not produce IFN-γ (data not shown).

To determine whether the recognition of LNCaP cells by PSGR-derived peptide-specific T cells is HLA-I restricted, we co-cultured LNCaP cells with PSGR-derived peptide-specific T cells in the presence of either anti-HLA-I mAb (W6/32) or control mAbs (HLA-II mAb or anti-CD19 mAb). As shown in Figure 3B, the cytotoxicity of these peptide-specific T cells was completely inhibited by the addition of anti-HLA-I mAb, but not by anti-HLA- II (HLA-DR) or a control mAb (anti-CD19), which suggests that the recognition of LNCaP cells by PSGR-derived peptide-specific T cells is HLA-I restricted.

## Discussion

It is well established that CD8^+^ T cells play a critical role in controlling tumor development and progression. Peptide epitopes derived from tumor-associated antigens (TAAs) can be recognized as antigens by T cells in the context of MHC-I molecules [32], [33]. Identification of TAAs and their peptides that are recognized by T cells are essential for the development of effective cancer vaccines.

The aim of the current study was to identify HLA-A2 binding PSGR-derived epitopes recognized by CD8^+^ T cells in PBMCs of healthy subjects and prostate cancer patients. Three different computer-based prediction algorithms including BIMAS, SYFPEITHI, and Rankpep were used to scan the PSGR protein sequence for HLA-A2 binding peptides based on the HLA-A2 binding motif. Only peptides that were predicted successfully by at least 2 out of 3 of the different computer-based prediction algorithms were included. Twenty-one 9mer or 10mer peptides were selected in this study according to this criterion. All these peptides were tested for their ability to stimulate PBMCs from either healthy subjects or prostate cancer patients to release IFN-γ. Of 21 peptides, three peptides frequently induced specific T cell responses in PBMCs obtained from either healthy subjects or cancer patients, and these peptide-specific T cells also recognized HLA-A2^+^ PSGR-expressing LNCaP cells, suggesting that these peptides are naturally processed by prostate cancer cells.

PSGR is a prostate tissue-specific gene with homology to the G protein-coupled odorant receptor gene family and it is specifically expressed in human prostate tissues [23]–[25]. The expression of PSGR is significantly higher in human prostate intraepithelial neoplasia and prostate tumors than normal tissues [25]. Intriguingly, although PSGR has been considered to be a novel target for prostate cancer immunotherapy, T cell epitopes derived from PSGR have not been identified. This is, to our knowledge, the first report to identify and characterize PSGR-derived epitopes recognized by CD8^+^ T cells. The identification of PSGR-derived epitopes recognized by T cells further validates PSGR as a promising target for the development of cancer vaccines.

Most TAAs are self-antigens [34], therefore, self-tolerance may occur in an attempt to protect the individual from the development of autoimmunity. This is considered to be a major obstacle in the induction of TAA-specific T cells capable of eradicating tumors *in vivo.* However, in our study, although PSGR is expressed in normal prostate tissue, immune tolerance against PSGR can be broken, since T cell responses against PSGR-derived epitopes were frequently detectable in PBMCs from either healthy subjects or prostate cancer patients.

A vast number of immunotherapy clinical trials based on vaccinations with tumor lysates, TAA proteins, TAA peptides and RNA or DNA encoding TAA have already been conducted. However, most of these trials have not achieved desirable results. One reason is that expression of these TAAs is heterogeneous among tumors from different patients and can vary even among metastases obtained from one patient [35], [36], thus immune escape may occur when the immunotherapeutic approach is only based on one TAA. To avoid immune escape, vaccine-based immunotherapeutic strategies that target several tumor antigens are essential for the development of successful cancer vaccines. Thus identification of additional prostate specific tumor antigens, such as PSGR, for T-cell-based immunotherapy is still needed, despite that a number of prostate specific tumor antigens including PSA [12], [13], PSCA [16], PSMA [17]–[19], PAP [20], Prostein [14], [15] and trp-p8 [21], have been identified in the last few years.

The FDA has recently approved a cancer vaccine, Sipuleucel-T, for the treatment of patients with advanced prostate cancer based on a phase III study [8]. Sipuleucel-T is prepared from autologous PBMCs containing antigen presenting cells that are incubated with a recombinant protein composed of a PAP linked to granulocyte-macrophage colony-stimulating factor (GM-CSF). Sipuleucel-T presumably works in part by augmenting PAP-specific CD8^+^ T cell responses, further demonstrating the importance of tumor antigen-specific CD8^+^ T cells induced by cancer vaccines. So far, Sipuleucel-T is the first cellular immunotherapeutic agent approved by the FDA to be used for the treatment of cancer patients. The FDA approval of Sipuleucel-T as a therapeutic cancer vaccine not only validates the efficacy of cancer immunotherapy, but also provides a strong impetus in the field of cancer immunology [37]. Therefore, identification and development of more novel TAAs including PSGR and peptide derivates recognized by CTLs is definitely essential to facilitate the development of effective cancer vaccines against prostate cancers as well as other types of cancers in the future.

Furthermore, the epitopes recognized by CD8^+^ T cells may be used as diagnostic tools to monitor peptide-specific CD8^+^ T cells in individuals during the course of immunization, thus identifying optimal time frames for immunization during treatment, including whether subsequent immunizations are needed in individuals when anti-tumor immunity declines.

In summary, we have identified three novel PSGR-derived CTL epitopes. Since PSGR expression is strongly up-regulated in human prostate cancers, PSGR-derived peptides may serve as diagnostic tools or immunotherapeutic targets of anticancer vaccines alone or in combination with other epitopes that are derived from other prostate-specific antigens.
